# Prognostic Nomograms Based on the Cirrhotic Severity Scoring for Preoperative Prediction of Long-Term Outcomes in Patients with HBV-Related Hepatocellular Carcinoma and Child-Pugh Grade A Liver Function

**DOI:** 10.1155/2022/7031674

**Published:** 2022-05-21

**Authors:** Jin Gu, Bin-yong Liang, Er-lei Zhang, Zhi-yong Huang

**Affiliations:** ^1^Hepatic Surgery Center, Tongji Hospital, Tongji Medical College, Huazhong University of Science and Technology, Wuhan, China; ^2^Department of Hepatobiliary and Pancreatic Surgery, The Second Affiliated Hospital, School of Medicine, Zhejiang University, Hangzhou, China

## Abstract

**Background:**

Cirrhotic severity scoring (CSS) is a noninvasive method that can predict histological severity of cirrhosis. This study is aimed at assessing the predictive value of CSS on long-term outcomes after curative hepatectomy for patients with hepatitis B virus- (HBV-) related hepatocellular carcinoma (HCC) and Child-Pugh grade A liver function and further developing novel nomograms to preoperatively predict posthepatectomy recurrence and survival.

**Methods:**

Consecutive patients who underwent curative hepatectomy for HCC between 2008 and 2014 were retrospectively studied. According to the CSS, patients were subclassified into 3 groups: no/mild, moderate, and severe cirrhosis. The impact of CSS on recurrence-free survival (RFS) and overall survival (OS) was assessed. Furthermore, RFS and OS nomograms were developed.

**Results:**

The 5-year RFS and OS rates were 36.1% and 62.8% in the no/mild cirrhosis group, compared with 28.4% and 56.2% in the moderate cirrhosis group, and 16.2% and 33.0% in the severe cirrhosis group. Long-term survival outcomes were significantly worse with the increment of cirrhotic severity. CSS, alpha-fetoprotein level, tumor size, tumor number, and macrovascular invasion were identified as independent predictors of both RFS and OS. Besides, albumin-bilirubin grade was an independent risk factor of OS not RFS. RFS- and OS-predictive nomograms based on these preoperative variables were built. For these 2 nomograms, the C-indexes were 0.696 and 0.732, respectively. Calibration curves exhibited good agreement between actual observation and nomogram prediction.

**Conclusions:**

CSS was a predictor for long-term outcomes in HCC patients after curative hepatectomy. The novel nomograms exhibited accurate preoperative prediction of posthepatectomy recurrence and OS.

## 1. Background

Hepatocellular carcinoma (HCC) is the sixth most common cancer and ranks the fourth leading cause of cancer-related mortality in the world [1]. Currently, hepatic resection remains the mainstay curative treatment option for HCC patients [2]. Improvements in surgical techniques and perioperative care have improved the safety of hepatectomy, and many studies have demonstrated that HCC patients diagnosed at intermediate and advanced stages can also benefit from hepatic resection [3, 4]. Nevertheless, the prognosis of HCC patients after curative hepatectomy remains unsatisfactory [5].

In China, most HCC patients were accompanied by cirrhosis [6], and cirrhosis is not a consistent entity in terms of histological changes [7, 8]. According to the fibrous septal thickness and nodule size, the Laennec staging system divides cirrhosis from mild to severe [7]. This staging system has been validated to be useful in predicting prognosis in HCC patients after hepatectomy [9, 10]. Although histological staging of cirrhosis was important for the surgical decision-making, a preoperative liver biopsy is invasive. In 2016, our previous research proposed a noninvasive approach called cirrhotic severity scoring (CSS) to evaluate histological severity of cirrhosis in HCC patients [11]. The CSS has been demonstrated to have a high degree of diagnostic accuracy in evaluating histological severity of cirrhosis [11, 12]. However, it remains unknown whether this method can be implemented to predict outcomes in HCC patients who underwent curative hepatectomy. Furthermore, previously established predictive models for HCC mainly focused on tumor-related features, which cannot fully explain the heterogeneity of prognosis in HCC patients who underwent hepatectomy with curative intent. Thus, evaluating the prognostic significance of noninvasive methods that can reflect histological severity of cirrhosis is reasonable for providing additional prognostic information for HCC patients.

Recently, nomograms have been developed for many tumor types and accepted as reliable tools to predict survival for patients with malignancies [13–16]. Since nomograms have been validated to provide more precise prediction when compared with the traditional staging systems for many cancers, they have been proposed as an alternative or even a new standard approach [17, 18].

This study is aimed at elucidating the prognostic significance of CSS in HCC patients after hepatectomy with curative intent. We also sought to construct novel prognostic nomograms that included CSS and other independent prognostic factors based on the data obtained before surgery to preoperatively predict the possibility of tumor recurrence and survival. In addition, we further compared the discriminatory powers of the nomograms with the existing classification systems, including the Barcelona Clinic Liver Cancer (BCLC) stage [19] and the Cancer of the Liver Italian Program (CLIP) score [20].

## 2. Methods

### 2.1. Patients

Between January 2008 and December 2014, a total of 636 patients with hepatitis B virus- (HBV-) related HCC and Child-Pugh grade A liver function who underwent curative hepatectomy at Tongji Hospital were included in the present study. Routine preoperative evaluation included serological examination, chest radiography, ultrasonography, endoscopy, and computed tomography (CT)/magnetic resonance imaging (MRI) of abdomen. The preoperative liver function was evaluated with the Child-Pugh system, liver biochemistry, and indocyanine green retention test. Tumor sizes and numbers were measured according to the preoperative imaging findings. This study was approved by the ethics committee of Tongji Hospital, Huazhong University of Science and Technology, China. Written informed consent for hepatic resection and further research was obtained from each patient.

### 2.2. Hepatectomy and Follow-Up

All operations were carried out under the general anesthesia and performed as previously described [21]. The abdominal cavity was carefully explored to determine the extent of local disease, extrahepatic metastases, and peritoneal seeding. During parenchymal transection, the intermittent Pringle maneuver was employed when necessary. Abdominal drainage was routinely placed.

Patients were followed up once a month within 6 months after hepatectomy and once every 2 months within 6 months to 2 years. Thereafter, patients were followed at a 3- to 6-month interval. At each follow-up visit, patients were routinely examined with serum alpha-fetoprotein (AFP), abdominal ultrasonography, and liver function tests. If tumor recurrence was suspected, further CT, MRI, bone scans, or positron emission tomography (PET) were performed. Patients who developed tumor recurrence were treated with repeat hepatectomy, ablative therapy, ethanol injection, transarterial chemoembolization, radiation, or sorafenib based on the recurrence pattern and liver function status. Overall survival (OS) was defined as the interval between the date of hepatectomy and the date of death or last follow-up. Recurrence-free survival (RFS) was defined as interval between the date of hepatectomy and the date of recurrence or the date of death or last follow-up if recurrence was not found.

### 2.3. Calculation of Cirrhotic Severity Scoring and Albumin-Bilirubin Grade

CSS was calculated according to 4 clinical parameters that are described in Table 1 [11]. The albumin-bilirubin (ALBI) score was calculated according to the following equation: 0.66 × log10 total bilirubin–0.085 × albumin. The ALBI grade was defined as grade 1 (score ≤ −2.60), grade 2 (score more than -2.60 and ≤-1.39), and grade 3 (score more than -1.39) [22].

### 2.4. Statistical Analysis

Statistical analysis was performed using SPSS 20.0 and R 4.0.0 (http://www.r-project.org). Continuous variables were described as mean with standard deviation, and categorical variables were described as numbers with percentages. Survival curves were presented using the Kaplan-Meier method and compared between groups using the log-rank test. Univariate and multivariate analyses for the prognostic factors were based on Cox's proportional hazards model. The hazard ratios (HR) and 95% confidence intervals (95% CI) were calculated.

The nomograms for RFS and OS were established based on the results of independent factors on multivariate analysis using the package of rms in R 4.0.0. The performance of the nomograms was measured by the concordance index (C-index) and calibration curves comparing the nomogram predicted versus the actually observed Kaplan-Meier estimates of survival probability. Internal validation was performed using bootstraps with 1000 resamples. The value of the C-index ranges from 0.5 to 1.0, with 0.5 indicating no discrimination and 1.0 indicating a perfect discrimination. The higher the C-index was, the more accurate the prognostic prediction would be [22]. Based on the constructed nomogram, each patient would obtain a nomogram's score for risk stratification of recurrence or postoperative mortality. Patients were then divided into different risk groups using the tertiles of the nomogram's scores as the cut-off points. A 2-sided *P* < 0.05 was considered statistically significant.

## 3. Results

### 3.1. Baseline Characteristics

The characteristics of patients are shown in Table 2. Among the 636 patients enrolled in the present study, 575 patients (90.4%) were male, and 61 (9.6%) were female, with a mean age of 49.3 ± 10.8 years. The liver function of all patients was classified as Child-Pugh A. Three hundred and fifty-two patients (55.3%) were classified as ALBI grade 1, and 284 (44.7%) as ALBI grade 2. The mean tumor diameter was 6.3 ± 3.7 cm (range, 1.0-22.0 cm). Five hundred and twenty-five patients (82.5%) had a single tumor, and 111 (17.5%) had multiple tumors. According to the CSS, 363 patients (57.1%) had no/mild cirrhosis, 175 (27.5%) had moderate cirrhosis, and 98 (15.4%) had severe cirrhosis.

### 3.2. Long-Term Survival Outcomes

**Figure 1:**
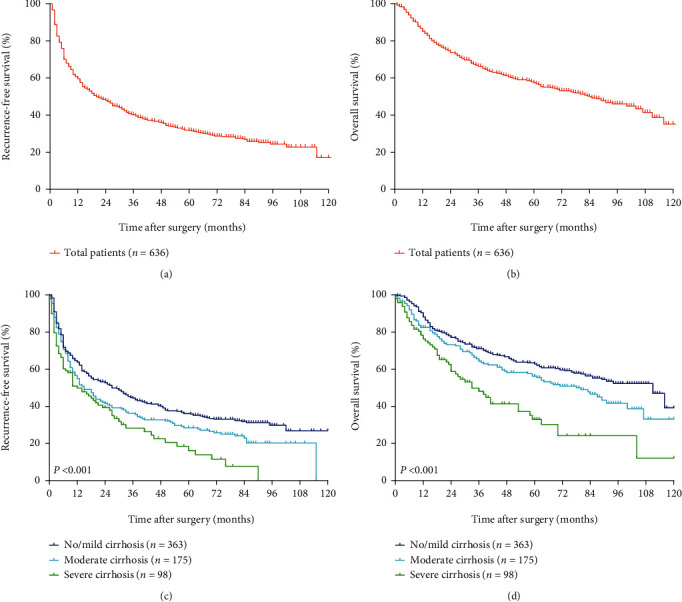
The Kaplan-Meier survival curves in the entire cohort and the groups stratified by cirrhotic severity scoring. (a, c) RFS curves. (b, d) OS curves. RFS: recurrence-free survival; OS: overall survival.

The 1-, 3-, and 5-year RFS and OS rates of the entire cohort were 59.3%, 40.0%, and 31.6%, respectively, and 85.3%, 66.1%, and 57.3%, respectively (Figures 1(a) and 1(b)).

In the group of patients with no/mild cirrhosis, the 1-, 3-, and 5-year RFS rates were 63.8%, 44.4%, and 36.1%, respectively, and the 1-, 3-, and 5-year OS rates were 88.2%, 71.1%, and 62.8%, respectively. In the group of patients with moderate cirrhosis, the 1-, 3-, and 5-year RFS rates were 55.0%, 36.2%, and 28.4%, respectively, and the 1-, 3-, and 5-year OS rates were 82.5%, 64.3%, and 56.2%, respectively. In the group of patients with severe cirrhosis, the 1-, 3-, and 5-year RFS rates were 49.9%, 28.2%, and 16.2%, respectively, and the 1-, 3-, and 5-year OS rates were 76.3%, 48.1%, and 33.0%, respectively (Figures 1(c) and 1(d)). Patients in the no/mild cirrhosis group had better RFS (*P* = 0.022 and < 0.001, respectively) and OS (*P* = 0.032 and < 0.001, respectively) rates than those in either the moderate or severe cirrhosis group. Furthermore, patients in the moderate cirrhosis group had better RFS (*P* = 0.042) and OS (*P* = 0.001) rates than those in the severe cirrhosis group.

### 3.3. Identification of Independent Predictors of Recurrence-Free and Overall Survival Based on the Preoperative Data

In an effort to analyze the independent predictors of RFS and OS on the basis of the preoperative data, all variables obtained before surgery were used for the Cox univariate and multivariate regression analyses. The variables selected included age, gender, alanine transaminase level, ALBI grade, prothrombin time, AFP level, CSS, tumor number, tumor size, and macrovascular invasion and vascular invasion (Table 3). Significant risk factors (*P* < 0.05) identified by univariate analysis were entered into the Cox multivariate analysis. The results showed that CSS, AFP level, tumor number, tumor size, and macrovascular invasion were independent risk factors of both RFS and OS. In addition, the ALBI grade was an independent risk factor of OS not RFS (Table 4).

### 3.4. Construction and Validation of the CSS-Based Nomograms for RFS and OS

**Figure 2:**
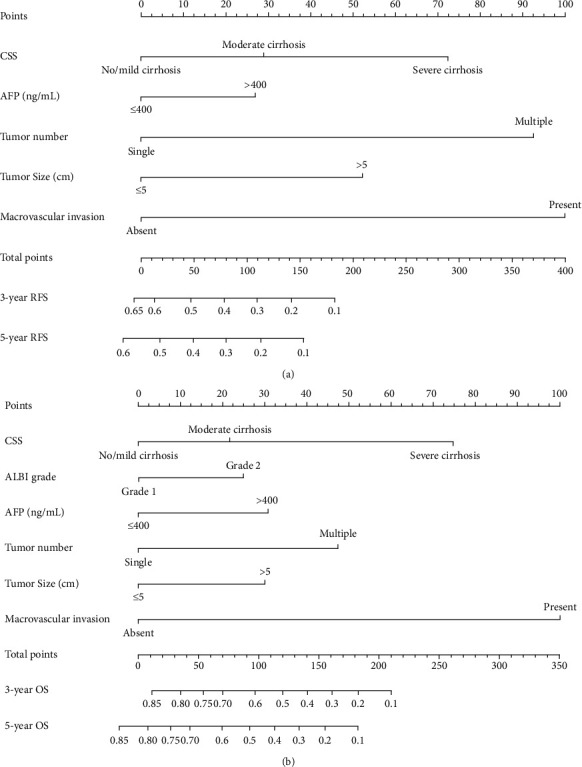
The preoperative nomograms for predicting RFS and OS in patients with hepatocellular carcinoma undergoing curative hepatectomy. (a) RFS predictive nomogram. (b) OS predictive nomogram. To use the nomogram, an individual patient's value is located on each variable axis, and a line is drawn upward to determine the number of points received for each variable value. The sum of these numbers is located on the total point axis, and a line is drawn downward to the survival axes to determine the likelihood of 3- and 5-year RFS, and 3- and 5-year OS. CSS: cirrhotic severity scoring; ALBI: albumin-bilirubin; AFP: alpha-fetoprotein; RFS: recurrence-free survival; OS: overall survival.

**Figure 3:**
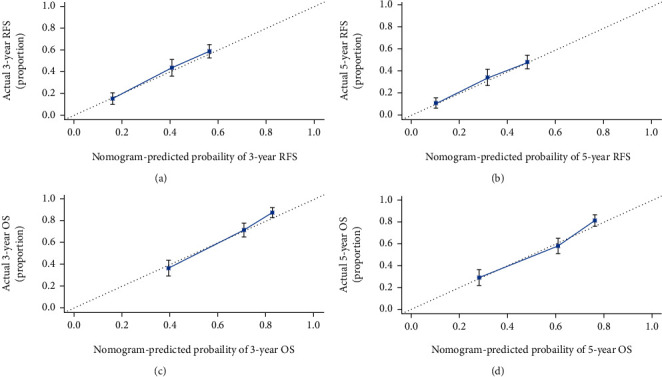
The calibration curves of RFS and OS based on nomogram prediction and actual observation. (a, b) The 3- and 5-year RFS rates. (c, d) The 3- and 5-year OS rates. The actual observation was plotted on the *y*-axis, and the nomogram-predicted probability was plotted on the *x*-axis. RFS: recurrence-free survival; OS: overall survival.

Preoperative nomograms that incorporated these independent predictors of RFS and OS on the basis of the preoperative data are shown in Figure 2. The C-indexes of the RFS and OS nomograms were 0.696 (95% CI, 0.671-0.721) and 0.732 (95% CI, 0.705-0.759), respectively, and with 1000 cycles of bootstrapping. Calibration curves based on the independent predictors are shown in Figure 3. There was good agreement between the actual observation and prediction by nomograms either of 3- and 5-year RFS or of 3- and 5-year OS.

### 3.5. Comparison of Discriminatory Powers of Nomograms with Conventional Staging Systems

The discriminatory powers of the RFS and OS nomograms were compared with BCLC stage and CLIP score. Our nomograms exhibited better discriminatory accuracy in predicting RFS than the competing models: its C-index was 0.696 (95% CI, 0.671-0.721), substantially higher than those of the BCLC stage (0.621 (95% CI, 0.599-0.643); *P* < 0.001) and CLIP score (0.641 (95% CI, 0.616-0.666); *P* < 0.001). In terms of OS, the C-index of our nomogram was 0.732 (95% CI, 0.705-0.759), significantly higher than those of the BCLC stage (0.647 (95% CI, 0.618-0.676); *P* < 0.001) and CLIP score (0.657 (95% CI, 0.628-0.686); *P* < 0.001).

### 3.6. Performance of Nomograms in Stratifying Risk of Patients

**Figure 4:**
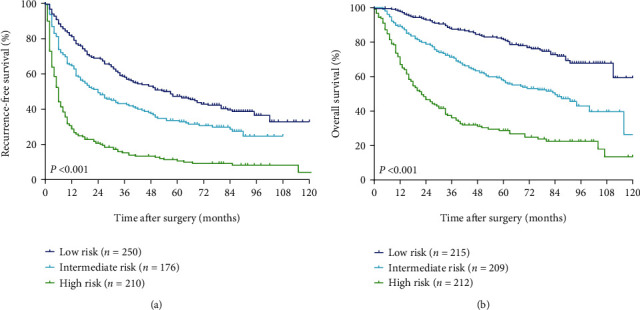
The Kaplan-Meier survival curves of different risk groups stratified by the tertiles of the preoperative nomogram's scores derived from the RFS and OS nomograms. (a) RFS curves. (b) OS curves. RFS: recurrence-free survival; OS: overall survival.

Based on the tertiles of RFS-nomogram's scores, patients could be classified into low-, intermediate-, and high-risk of recurrence. The 5-year RFS rates of these 3 groups were 47.3%, 33.5%, and 10.6%, respectively (*P* < 0.001; Figure 4(a)). According to the tertiles of OS nomogram's scores, patients could also be stratified into low-, intermediate-, and high-risk of postoperative mortality. The 5-year OS rates of these 3 groups were 81.4%, 57.5%, and 28.7%, respectively (*P* < 0.001; Figure 4(b)).

## 4. Discussion

According to current guidelines, liver resection is mainly recommended in HCC patients with Child-Pugh grade A, but selected patients with Child-Pugh grade B liver function can be considered for laparoscopic liver resection as previously described [23]. In this study, we included 636 HCC patients with Child-Pugh grade A liver function to demonstrate that CSS was independently associated with HCC recurrence and OS in HCC patients after curative hepatectomy. This indicated that such a noninvasive method used for evaluating cirrhotic severity could provide an important prognostic reference for HCC patients. We subsequently built RFS and OS nomograms that could be used for individualized evaluation of RFS and OS in patients with HCC treated with hepatectomy with curative intent. This is helpful for preparing HCC patients for treatment planning and postoperative follow-up.

Recently, several studies have demonstrated that histological severity of cirrhosis adversely affects the long-term outcomes in HCC patients after hepatectomy [9, 10]. However, many previously established prognostic models for HCC were largely based on tumor-related features, and the evaluation of cirrhotic severity was neglected to some extent. In the current surgical strategy for HCC, the Child-Pugh system is the most widely used index for assessing liver function status. However, the Child-Pugh system focused on liver function and could not reflect histological severity of cirrhosis [12, 24]. Besides, several studies revealed that this system was not an ideal prognostic indicator of survival in patients with HCC [25, 26]. Thus, effective noninvasive methods to evaluate cirrhotic severity are urgently required, and a determination of whether these methods can refine the existing survival predictive models is necessary. In the present study, we used a noninvasive approach named CSS to predict the outcome of HCC patients treated with curative hepatectomy. The CSS was previously developed to predict histological severity of cirrhosis in HCC patients [11]. It is based on 4 variables that are easily obtained through preoperative examinations. This noninvasive method has been found to be significantly correlated with histological severity of cirrhosis [11, 12]. The present study further demonstrated that CSS was strongly associated with RFS and OS in HCC patients who underwent curative hepatectomy. Multivariate analysis identified CSS as an independent adverse prognostic factor of RFS and OS.

In addition to CSS, our research revealed that tumor size, tumor number, AFP level, and macrovascular invasion were independent risk factors associated with both RFS and OS by the multivariate Cox regression analysis. The size and number of tumor have included in many HCC prognostic systems [19, 20], since these parameters can reflect the extent of tumor involvement. In this study, tumor size was classified by the cut-off value of 5 cm based on the Milan criteria. AFP, as a tumor marker, is widely used for the diagnosis of HCC [27]. Serum AFP level above 400 ng/mL was also reported to predict poor RFS and OS in patients with HBV-associated HCC after hepatectomy [28]. Besides, macrovascular invasion has been recognized as a critical and adverse prognostic factor for long-term survival in HCC patients after hepatic resection [29]. The ALBI score was specifically developed to evaluate liver function in HCC patients [30]. The present study found that patients with ALBI grade 1 had better prognosis that those with ALBI grade 2 after hepatectomy with curative intent, consistent with previous findings [30, 31]. Multivariate analysis further revealed that ALBI grade was an independent risk factor of OS.

In this study, we constructed RFS and OS nomograms to predict RFS and OS based on the preoperative data in HCC patients undergoing curative hepatectomy. The 2 nomograms constructed in the present study had unique features, which integrated both tumor characteristics and cirrhotic severity. The C-indexes were 0.696 and 0.732 of the nomograms to predict RFS and OS, which were significantly higher than those of the BCLC stage and CLIP score. Notably, the calibration plots for RFS and OS prediction well matched the idealized 45° line, confirming the predictive accuracy of the 2 nomograms. In addition, the prognostic nomograms based on the preoperative data could divide HCC patients into 3 distinct prognostic groups with different risks of tumor recurrence or mortality. Therefore, the 2 CSS-based nomograms could be used to predict RFS and OS on an individual basis for HCC who underwent curative hepatectomy.

This study has a few limitations. First, this was a retrospective study taking place in a single center, which may result in a selection bias. Second, due to the retrospective nature of this study, the records of HBV-DNA cannot be found in the computerized database for all patients. Therefore, we did not analyze the impact of positive HBV-DNA on the long-term outcomes in HCC patients after curative hepatectomy. Third, we only used our population to build 2 nomograms, and our findings were not verified in a validation cohort. Thus, multicenter and prospectively designed studies ought be conducted to further validate the reliability of these 2 nomograms. Fourth, the proportion of alcohol-related cirrhosis was small, which was different from Western countries. In addition, this study was of retrospective nature. Some data regarding the alcohol intake history were missing. Therefore, we did not analyze the prognostic impact of alcohol intake.

## 5. Conclusions

The present study revealed that CSS was associated with tumor recurrence and OS in HBV-related HCC patients after curative hepatectomy. Furthermore, we have developed 2 nomograms based on the preoperative data to predict posthepatectomy recurrence and OS. These nomograms achieved good predictive performance and discriminatory ability.

## Figures and Tables

**Table 1:** Cirrhotic severity scoring for staging cirrhosis.

Variables	Score		
	0	1	2
Platelet count (10^9^/L)	≥100	70-100	<70
Portal vein diameter (cm)	<1.2	1.2-1.4	>1.4
Splenic thickness (cm)	<4.0	4.0-5.0	>5.0
Varicosity	No	Mild	Moderate/severe
	No/mild cirrhosis	Moderate cirrhosis	Severe cirrhosis
Cirrhotic severity scoring	0~1	2~3	≥4

**Table 2:** Baseline characteristics.

Characteristics	Value
Age, years	49.3 ± 10.8
Gender, *n* (%)	
Male	575 (90.4%)
Female	61 (9.6%)
Alanine aminotransferase, U/L	40.0 ± 37.4
Albumin, g/L	39.6 ± 4.7
Total bilirubin, *μ*mol/L	13.5 ± 5.8
ALBI grade	
Grade 1	352 (55.3%)
Grade 2	284 (44.7%)
Prothrombin time, seconds	13.2 ± 1.6
AFP, *n* (%)	
≤400 ng/mL	372 (58.5%)
>400 ng/mL	264 (41.5%)
Platelet count, 10^9^/L	141.8 ± 70.0
Portal vein diameter, cm	1.2 ± 0.2
Splenic thickness, cm	3.9 ± 0.9
Varicosity, *n* (%)	
No	554 (87.1%)
Mild	45 (7.1%)
Moderate	15 (2.4%)
Severe	22 (3.5%)
CSS, *n* (%)	
No/mild cirrhosis	363 (57.1%)
Moderate cirrhosis	175 (27.5%)
Severe cirrhosis	98 (15.4%)
Tumor size, cm	6.3 ± 3.7
Tumor number, *n* (%)	
Single	525 (82.5%)
Multiple	111 (17.5%)
Macrovascular invasion, *n* (%)	
Absent	560 (88.1%)
Present	76 (11.9%)
BCLC stage, *n* (%)	
0/A	474 (74.5%)
B	82 (12.9%)
C	80 (12.6%)
CLIP score, *n* (%)	
0	293 (46.1%)
1	231 (36.3%)
2	72 (11.3%)
3	27 (4.2%)
4	13 (2.0%)

ALBI: albumin-bilirubin; AFP: alpha-fetoprotein; CSS: cirrhotic severity scoring; BCLC: Barcelona Clinical Liver Cancer; CLIP: Cancer of the Liver Italian Program.

**Table 3:** Univariate analysis of risk factors influencing recurrence-free and overall survival based on the preoperative data.

Variables	Recurrence-free survival		Overall survival	
	HR (95% CI)	*P*	HR (95% CI)	*P*
Age, years				
>60 vs. ≤60	0.990 (0.769-1.274)	0.936	1.180 (0.878-1.586)	0.272
Gender				
Male vs. female	1.001 (0.726-1.380)	0.996	0.893 (0.605-1.319)	0.569
Alanine aminotransferase, U/L				
>40 vs. ≤40	1.331 (1.095-1.618)	0.004	1.362 (1.072-1.731)	0.012
ALBI grade				
Grade 2 vs. grade 1	1.374 (1.141-1.654)	0.001	1.588 (1.262-1.998)	<0.001
Prothrombin time, seconds				
>14 vs. ≤14	1.074 (0.875-1.318)	0.495	1.046 (0.808-1.352)	0.735
AFP, ng/mL				
>400 vs. ≤400	1.442 (1.196-1.738)	<0.001	1.647 (1.309-2.072)	<0.001
CSS				
Moderate cirrhosis vs. no/mild cirrhosis	1.277 (1.033-1.578)	0.024	1.329 (1.021-1.730)	0.035
Severe cirrhosis vs. no/mild cirrhosis	1.676 (1.290-2.177)	<0.001	2.279 (1.672-3.107)	<0.001
Tumor number				
Multiple vs. single	2.470 (1.974-3.089)	<0.001	2.230 (1.703-2.921)	<0.001
Tumor size, cm				
>5 vs. ≤5	1.763 (1.461-2.128)	<0.001	1.825 (1.443-2.309)	<0.001
Macrovascular invasion				
Present vs. absent	2.952 (2.270-3.841)	<0.001	4.690 (3.497-6.289)	<0.001

ALBI: albumin-bilirubin; AFP: alpha-fetoprotein; CSS: cirrhotic severity scoring.

**Table 4:** Independent prognostic factors for recurrence-free and overall survival by multivariate analysis.

Variables	Recurrence-free survival		Overall survival	
	HR (95% CI)	*P*	HR (95% CI)	*P*
Alanine aminotransferase, U/L				
>40 vs. ≤40	1.171 (0.959-1.430)	0.121	1.182 (0.926-1.510)	0.180
CSS				
Moderate cirrhosis vs. no/mild cirrhosis	1.271 (1.022-1.580)	0.031	1.340 (1.023-1.756)	0.034
Severe cirrhosis vs. no/mild cirrhosis	1.772 (1.353-2.319)	<0.001	2.666 (1.934-3.676)	<0.001
ALBI grade				
Grade 2 vs. grade 1	1.191 (0.984-1.440)	0.073	1.385 (1.095-1.751)	0.007
AFP, ng/mL				
>400 vs. ≤400	1.285 (1.055-1.566)	0.013	1.516 (1.185-1.940)	0.001
Tumor number				
Multiple vs. single	2.186 (1.743-2.741)	<0.001	1.859 (1.411-2.449)	<0.001
Tumor size, cm				
>5 vs. ≤5	1.496 (1.217-1.840)	<0.001	1.448 (1.117-1.877)	0.005
Macrovascular invasion				
Present vs. absent	2.302 (1.753-3.022)	<0.001	3.720 (2.728-5.072)	<0.001

ALBI: albumin-bilirubin; AFP: alpha-fetoprotein; CSS: cirrhotic severity scoring.

## Data Availability

The datasets used and analyzed during the current study are available from the corresponding author on reasonable request.
